# A Survey on Mobility Support in Wireless Body Area Networks

**DOI:** 10.3390/s17040797

**Published:** 2017-04-07

**Authors:** Beom-Su Kim, Kyong Hoon Kim, Ki-Il Kim

**Affiliations:** 1 Department of Informatics, Gyeongsang National University, Jinju 52828, Korea; bumsou10@naver.com (B.-S.K.); kikim@cnu.ac.kr (K.H.K.); 2 Department of Computer Science and Engineering, Chungnam National University, Daejeon 34134, Korea

**Keywords:** wireless body area network, mobility model, posture modeling, communication protocols, energy efficiency

## Abstract

Wireless Body Area Networks (WBANs) have attracted research interests from the community, as more promising healthcare applications have a tendency to employ them as underlying network technology. While taking design issues, such as small size hardware as well as low power computing, into account, a lot of research has been proposed to accomplish the given tasks in WBAN. However, since most of the existing works are basically developed by assuming all nodes in the static state, these schemes therefore cannot be applied in real scenarios where network topology between sensor nodes changes frequently and unexpectedly according to human moving behavior. However, as far as the authors know, there is no survey paper to focus on research challenges for mobility support in WBAN yet. To address this deficiency, in this paper, we present the state-of-the-art approaches and discuss the important features of related to mobility in WBAN. We give an overview of mobility model and categorize the models as individual and group. Furthermore, an overview of networking techniques in the recent literature and summary are compiled for comparison in several aspects. The article also suggests potential directions for future research in the field.

## 1. Introduction

Healthcare always remains the highest priority. In addition, recently several network technologies, such as wireless ad hoc and sensor networks, enable us to use their approaches for healthcare application. Small sized sensor nodes around the body are involved in gathering medical information and transmitting them toward the sink node, which is supposed to communicate with nodes in Internet. With the help of such technologies, both patients and doctors can be updated anytime and anywhere so they can handle emergency situations. Furthermore, a number of smart applications have emerged in several domains including telemedicine and data gathering for scientific research [1,2,3].

However, the existing network technologies such as pure ad hoc and Wireless Sensor Networks (WSNs) are not feasible to meet specific requirements; therefore, special Wireless Body Area Networks (WBANs) have been proposed and attracted interests of researchers in the community. Even though communication between sensor nodes is a big, common concern in both WBANs and WSNs, there are also fundamental differences between them in terms of network environments and requirements. For instance, (i) varying bandwidth of communications through body rather than space; (ii) energy dependent heterogeneous nodes; (iii) strict constraints for power consumption; and being (iiii) harmless to the body tissues are unique characteristics of WBAN compared to WSN; therefore, new hardware and software are proposed to deploy various WBAN applications in the real world. Here, we review one of the software architectures. Fortino et al. [4] proposed efficient software framework and signal-processing application. Signal Processing In Node Environment (SPINE) provides Application Programming Interfaces (APIs). Therefore, it is reasonable to define SPINE as middleware running between coordinator (sink node) and sensor nodes. Furthermore, platform independent communication protocol layer in SPINE contains an abstract layer where IEEE 802.15.4 and Bluetooth are implemented. In more detail, SPINE consists of two main components: SPINE node and Coordinator. SPINE node is implemented by the embedded programming language and SPINE Coordinator by Java. SPINE node is composed of four interacting components, sensor node manager, communication block, sensing management and signal processing. They control all relevant functions to manage duty cycle, various timers and communications. SPINE Coordinator provides APIs for six main functions and four states. With the help of SPINE, each function is used to build rapid prototypes for WBAN nodes. As an extension of SPINE, Collaborative SPINE (C-SPINE) [5] was proposed to improve the SPINE by employing multiple coordinators called Base Station (BS) and information exchange with the help of Over-The-Air (OTA) protocol in inter-Body Sensor Networks (BSNs). C-SPINE operates in the same way as SPINE for non-collaboration applications. On the other hand, in the case of collaboration application, corresponding service is activated by following procedures, BSN service selection and activation, application-specific service, BSN proximity detection protocol and BSN service discovery protocol, which discovers neighbor BSs after exchanging lists of available services between them.

Besides software frameworks, there are several good survey papers [6,7,8,9] to address the key technologies as well as research challenges, such as security and low power computing, and these present further research trends. Especially, Ghamari et al. [9] emphasized the communication technique for rapid development and deployment of WBAN systems that support remote monitoring systems in residential environments. Furthermore, multi-sensor fusion in BSN-based human activity recognition was addressed in [10,11]. Sensor fusion strategies in BSNs were explained with domains of emotion recognition and general-health. However, even though they provide an overview of recent approaches in WBAN, there is no acceptable survey paper to focus on mobility support in WBAN yet. The sensors embedded or implanted in the body naturally have mobility according to human movement or specific behavior. Since such movements show different patterns with other typical mobility models in the several aspects, communication protocols for WBAN need to be developed to reduce the negative impact of movements. For example, Imran et al. studied the effect of critical nodes in mobile ad hoc networks. In order to remove negative effects such as network partition, it is necessary to identify the critical nodes in an efficient way. To achieve this goal, unlike the previous works demanding periodical updating for whole topology information, a Localized Algorithm for Segregation of Critical/Non-critical nodes (LASCNN) [12] was proposed to find the critical nodes with the partial information within a few hops. Moreover, Imran et al. [13] introduced an Application-centric Connectivity Restoration (ACR) algorithm aiming at minimum recovery time and overhead to restore connectivity for mission-critical application. Since connectivity is mostly dependent on critical nodes in Wireless Sensor and Actor Networks (WSAN), algorithms to identify and monitor critical nodes, and select backup nodes in the case of critical nodes’ failure were introduced. As a primary strategy, a node with the highest degree in an overlapped coverage area is set to be a critical node while the backup node is selected by taking multiple attributes, neighbor position, application-level interests, connectivity and overlapped coverage. When a failure is detected by a primary monitoring function for critical nodes, one of the post-failure recovery procedures is performed according to current critical and backup node status.

In conformity with mobility properties for WBANs and similarities between WSNs and WBANs, we can expect that performance of WBANs is also affected by nodes’ movement significantly. Nevertheless, the mobility issue is not highlighted in the above survey papers for WBAN, while there are many good survey and research papers to focus on mobility model and simulation tools in WSN. Motivated by this analysis, in this article, we survey the effects of mobility models in the design of WBANs and group them according to the number of moving nodes. Moreover, we review recent advances related to mobility for WBAN in networking techniques and present analysis with comparison of methods aforementioned. Finally, we conclude this article with a discussion of potential open issues.

## 2. Mobility Support in WBANs

There may be several ways to categorize the existing schemes for mobility support in WBANs. Among the possible ways of grouping, we focus on two principles: what is the research objective and how many nodes move in the same group. Currently, there are two major research topics to support mobility in WBAN. One is analytic (mathematical) modeling of mobility patterns and the other is networking technology to support mobility. The former is to extract relationships between moving nodes and then make them regular patterns. On the other hand, the latter is to define communication protocol and network architecture to handle topology change caused by human behavior.

The other applicable rule to grouping is the number of moving nodes with the same pattern. By applying this rule, the mobility model can be categorized as individual and group. Individual mobility model represents a person’s moving pattern such as walking, standing, lying and sitting. On the other hand, the group mobility model means the pattern that at least two sensor nodes belong to a group where every node follows a logical center (group leader) that determines the group’s motion behavior. Furthermore, as one more step for categorization, each mobility pattern can be divided into general and specific purposes according to movement place. While a general purpose model does not target any specific application, specific ones aim to model group pattern depending on applications in places such as hospitals.

**Figure 1:**
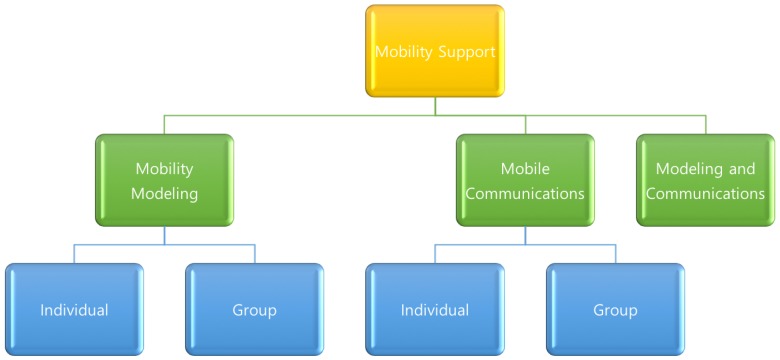
Categorization of mobility support for research objectives in wireless body area networks.

**Figure 2:**
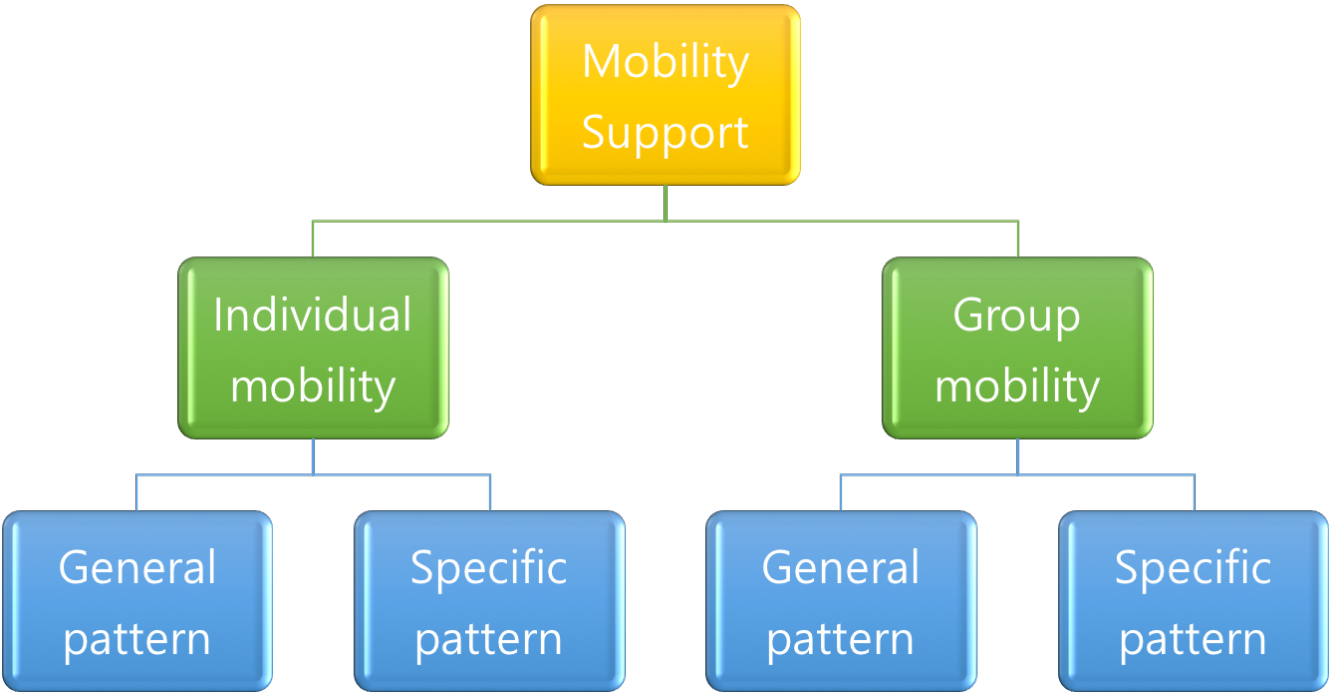
Categorization of mobility support for human groups in wireless body area networks.

Based on analysis for categorization, here we illustrate the result of possible categorization in both Figure 1 and Figure 2, while research objectives are the primary rule for categorization in Figure 1 and the mobility model in Figure 2. In this paper, we describe each scheme by giving higher priority to human behavior according to mobility model than research objective since clear classification and comparison between schemes are available. In this categorization, mobility model and network technology are explained, respectively.

## 3. Mobility Model and Communication Protocol

In this section, we briefly introduce the related schemes with goals and outstanding features. In addition, some outstanding schemes in each section are explained with the figure for better understanding.

### 3.1. Individual Mobility

Individual mobility scheme represents model and communication protocols for a person’s movement. This means that movement is mostly dependent on posture, such as walking, standing, sitting, running and so on. As for this mobility, general mobility assumes movement according to a person’s ordinary activity, while specific mobility does extraordinary movement with limitations in specific places such as hospitals. Of two mobility patterns, a lot of research assumes general mobility patterns rather than specific mobility since the latter demands high complexity for modeling. In this section, we present the mobility model and related networking technology.

#### 3.1.1. Mobility Model

Until now, most of the research for the mobility model focuses on single node mobility describing a person’s movement. The well-known and cited models include Random Walk, Random Waypoint and Random Direction that belong to the memory-less model [14]. On the other hand, Random Gauss–Markov Mobility [15] was proposed to get the value of speed and direction according to Markovian property. However, these models cannot be directly applicable for WBAN because they are intended to generate movement trace for each node, respectively. Thus, it is not feasible to produce relative position information of multiple sensor nodes on the body. To defeat this deficiency, Sandhu et al. [16] introduced detailed posture models for standing, sitting, laying, walking and running. As an example, sitting is modeled by posture on the chair. In this case, trunk of the human body shows little movement while larger dynamics are observed for human arms and legs. By this observation, they estimated the position of each sensor on knees and elbows. In the same way, mobility patterns for walking, running, and laying are collected by the distance and direction. In addition to each behavior modeling, selection between posture patterns is modeled by Markov chain, which presents the probability of posture change from one state to another. Also to study of the new mobility model, they analyzed the impact of mobility for performance from the aspect of energy consumption, delay and path loss.

#### 3.1.2. Protocols for General Mobility

Most of the protocols to support general mobility operate in either layer 3 or layer 2. For the layer 3 approach, flooding is likely to be considered in WBANs due to simple operation and low computational overhead. In addition, typical ad hoc networks protocols can support mobility in proactive and reactive ways. However, communication between sensor and sink node is mostly bounded by a few hops where a node’s mobility speed is lower than ad hoc networks; therefore, original ad hoc protocols intended to support a large number of hops at high speed need to be extended or newly developed to meet requirements of applications. Based on the above mentioned reason, modified flooding is desirable due to significant energy consumption. On the other hand, as a layer 2 approach, Time Division Multiple Access (TDMA) based Medium Access Control (MAC) protocols were proposed to meet real-time requirements and low overhead instead of high throughput where the amount of periodic reporting is overwhelming the event-driven in WBANs. In this section, we review the technical issue and present the recent advances in mobility support for WBAN.

Usually, multiple copies of packets are broadcasted to all nodes to establish routes between sink and sensor nodes in typical flooding schemes. As a next step, a route with minimum end-to-end delay is selected as the main path. However, due to path loss caused by topology partitioning in WBAN, Quwaider et al. proposed On-Body Store and Flood Routing (OBSFR) [17], which is based on the store and forward principle. To implement OBSFR, each packet carries a unique identifier which consists of a pair of (source ID, sequence number), and list of all node’s ID along the path from source and corresponding node. When a node receives a packet, it checks whether this packet is already received or not. If this packet already arrived before, it is discarded. Otherwise, the packet is stored in the buffer. The stored packet will be rebroadcasted when a node encounters any new node that does not possess a list of node IDs. Upon being rebroadcasted, the packet is removed from the buffer to make additional empty space. Even though OBSFR was proposed to support mobility in WBAN, OBSFR can be applicable to any networks, given that flooding is not controlled by any specific parameter for WBAN.

A similar approach to OSBFR was introduced in Probabilistic Routing with Postural Link Costs (PRPLC) [18]. The major distinct between OSBFR and PRPLC is the forwarding scheme. A packet stored in a node’s buffer is forwarded to a node which has a higher probability to meet the sink node than the current node. With the help of a new forwarding scheme in PRPLC, higher resource utilization is observed than OBSFR because the packet is not forwarded to all nodes. Furthermore, end-to-end delay is also affected by reducing the number of intermediate hops. In order to evaluate the meeting probability for sink nodes, PRPLC introduced a new metric: Link Likelyhood Factor (LLF). After each node calculates LFF for sink nodes, a node uses a HELLO message to carry its LFF value to neighboring nodes. When a node is connected with a sink node directly, a packet at the buffer is delivered. Otherwise, it can be either delivered to the next node or kept in the current node. The former happens when a node meets any node with a higher LLF value while the latter occurs when no higher LLF value is found in neighboring nodes.

Distance Vector Routing with Postural Link Costs (DVRPLC) [19] was proposed to reduce end-to-end delay by reducing waiting delay at the intermediate node. Even though DVRPLC is based on store and forward like OBSFR and PRPLC, there are slight differences in operation among them. DVRPLC uses a distance vector algorithm to compute accumulated cost of path toward the sink instead of a node’s value. Path cost between a node and sink node is computed by taking all neighbor nodes’ Link Cost Factor (LCF) value that is exchanged through a HELLO message. While LLF in PRPLC represents a node’s meeting probability to sink node, LCF totals all of the nodes’ LLF along the path from source to sink node. Based on LCF value, a similar forwarding policy to PRPLC is applied. If a node is within the sink node coverage, a packet is delivered directly. If not, a node *i* selects *k* with the least LCF value among the neighboring nodes and then forwards packet to *k*. When forwarding procedure is done, LCF value between *i* and sink is updated by adding two LCF values, that is, LCF between *i* and *k* and *k* and sink node. However, if there is no available node to satisfy the two conditions above, the packet is kept in the buffer until encountering a node satisfying the forwarding policy.

Opportunity Routing [20] was proposed to introduce relay nodes that are intended to overcome Line of Sight (LOS) limit in WBANs. They assume that relay function is embedded into all sensor nodes while the sink node is on the wrist. A sink node is not within LOS transmission path when a man takes his arms back. In this situation, a data packet is sent to relay node and then transmitted to sink node consequently. Detailed operating procedures for Opportunity Routing are as follows. As the first step, each node sends a Request to Send (RTS) message to the sink node. If a sink node is located in LOS, it sends an ACKnowledgement(ACK) message. Upon receiving an ACK message, a node sets up direct communication. However, if a node cannot receive an ACK message within the predetermined period, it becomes aware of the nonexistence of the node in LOS. In this case, a node sends a WAKE UP message to the relay node to turn its status to Receive Ready. Followed by the above procedures, a connection between a node and sink node via relay node is established. At the next step, a sink node sends a Receive ACK (RACK) message to the source node via a relay node. This procedure is repeated iteratively until a sink node does not get RACK message. Another approach to deploy a relay node is Relay based Thermal aware and Mobile Routing Protocol (RTM-RP) [21]. In this work, two types of mobility models are concerned: static and mobile set. The former refers to slow movement such as sitting, laying, and standing, whereas the latter includes fast movement such as walking and running. There are three time slots: Random Access Period (RAP) slotted Carrier-Sense Multiple Access (CSMA), Relay Buffer TDMA and Relay DATA TDMA. In RAP Slotted CSMA, each node tries to occupy the same channel as CSMA. A relay node exchanges buffered information with relay nodes in Relay Buffer TDMA. Based on obtained information during the Relay Buffer TDMA time slot, each relay node can predict the starting time and required number of slots. At last, a relay node transmits the data in buffer in the Relay DATA TDMA period. In addition to posture modeling, Sandhu et al. [14] studied a relay node based scheme which consists of initialization, forwarder selection, scheduling, and data transmission phase. At the initialization phase, a sink node broadcasts the HELLO message carrying the location of sink, location of neighbors, and all possible route information to the sink. After each node gets this HELLO message, the routing table is updated with the obtained information. Furthermore, each sensor involved in one of the two groups, *A* and *B*, according to distance to the sink node, selects a forwarder in the next phase. The forwarder decides the time slot for the child node in the TDMA scheme, which contributes to reducing energy consumption and collision. Finally, at the data transmission phase, each node transmits data to the forwarder during the allocated time slot. The forwarder aggregates the received data from many child nodes and forwards them to the sink node.

Another approach to introducing a link quality matrix was proposed in Prediction to enable Secure and Reliable routing (PSR) [22]. PSR is prediction based routing protocol to prevent data injection attack. To perform routing procedure based on the prediction model, each node builds backbone links with fixed neighbors to maintain constant distance in spite of body gestures. This new backbone link is updated with link quality. Based on the prediction model, the next hop is selected if it has the highest link quality in the current time slot as well as the previous one. However, whenever the prediction model is not available in the case of the random mobility model, a packet is transmitted over the shortest path tree on the backbone link.

In addition to the delay issue, temperature and amount of energy are taken as routing cost in Energy efficient Thermal and Power Aware (ETPA) [23], which performs a forwarding task under the store and forward principle. Instead of delay or link information, the HELLO message containing a node’s temperature and remaining energy is exchanged between neighbor nodes. These values are used to determine a node’s cost function by taking received power as well as neighbor’s temperature and energy level in a HELLO message. A node either sends a packet to one of neighbor nodes with a minimum cost value or keeps the packet in the buffer only if there is no suitable node to forward. Additionally, a packet is discarded when hop count of packet exceeds maximum hop count. This procedure continues until a packet is eventually delivered to the sink node.

**Figure 3:**
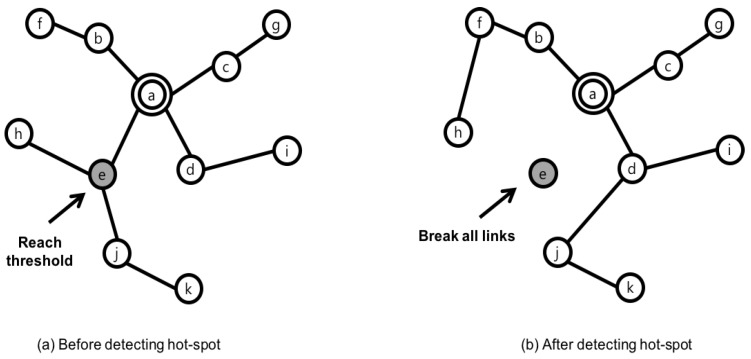
Hot spot by Mobility-supporting Adaptive Threshold-based Thermal-aware Energy-efficient Multi-hop ProTocol.

**Figure 4:**
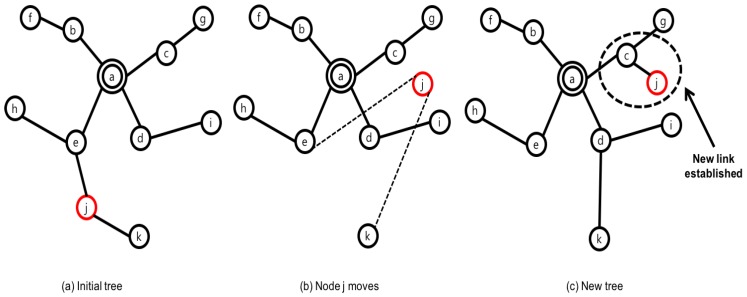
Node movement by Mobility-supporting Adaptive Threshold-based Thermal-aware Energy-efficient Multi-hop ProTocol.

As far as the authors are aware, the Mobility-supporting Adaptive Threshold-based Thermal-aware Energy-efficient Multi-hop ProTocol (M-ATTEMPT) [24] is the most applicable protocol for mobility in WBANs. It is a temperature aware protocol under tree structure. Whenever a person changes his/her gesture, the tree is rebuilt by a node’s joining to the new parent node. In M-ATTEMPT, a sink node locates at the center of body and the nearest node from a sink node is set to the parent node, which is root of the tree. When a node changes location, a larger amount of energy will be consumed if the current tree path is maintained. To prevent this unsuitable waste, a node connects to the new parent node after disconnecting from the link. Through these procedures, a new tree is constructed and a data packet is delivered along the links on the tree. Furthermore, M-ATTEMPT provides two major functions to handle hot spot and mobility at the same time as adjusting the tree. Figure 3 shows the procedure to prevent the hot spot. In this example, node e reaches the temperature threshold due to the forwarding task. Whenever each node becomes the hot spot, the parent node disconnects all links from child nodes during the next few rounds. When a node’s temperature status turns normal, a link is reestablished. If a hot spot parent node receives a data packet from the child, it returns the data packet to the child. If this case happens, the child node marks its parent as a hot spot in order to not deliver the packet next time. The mobility function provided by M-ATTEMPT is illustrated in Figure 4. If a child node moves to another place, it disconnects the link to the parent node in order to reduce the communication overhead. Upon being disconnected, a node searches the nearest parent node and rebuilds the tree dynamically by sending a joint-request message. These sequential procedures are explained in order of from Figure 4a–c.

IM-SIMPLE [25] is extended from the original Stable Increased-throughput Multi-hop Protocol for Link Efficiency (SIMPLE) protocol to take mobility into account. Its objective is to improve reliability and stability as well as achieve low energy consumption and high throughput by defining new cost function that takes a node’s residual energy as well as its relative distance. Each sensor node delivers a packet to a sink node via a forwarder node which is set to one with maximum residual energy and minimum distance. However, data delivery via a forwarder node may lead to rapid energy consumption on the forwarder. When energy levels of the forwarder node is lower than the threshold, a node sends a packet to the sink node directly. The mobility in IM-SIMPLE operates the same as M-ATTEMPT by finding a new forwarder node according to the position of the node. Another protocol to take link stability and remaining energy was proposed in [26]. Unlike the typical routing algorithm to focus on the path toward the sink node, the routing algorithm between multiple body coordinator and static coordinator was studied. That is, the proposed scheme handles inter-BAN and beyond-BAN communications. According to human mobility, topology changes frequently. To determine the next hop in unstable topology, energy and link stability are employed. In particular, link stability is computed by current node position, direction of movement and speed. In order to compute link stability between node *i* and *j*, node *i* periodically sends a message and gets the coordinate information of node *j*.

Unlike store and forwarding scheme for mobility in WBAN, Kim et al. proposed Mobility-Based Temperature-Aware Routing protocol (MTR) [27] that is based on store and carry, which is main principle of Delay Tolerant Networks (DTNS). Store and carry is used to re-route a packet when a packet is likely to be transmitted to a hot spot. This is to prevent the hot spot as well as packet loss by carrying this packet on mobile node instead of dropping. In MTR, the next hop is set to one of either low temperature or high probability to meet the sink node.

Until now, we have presented the overview of communication protocols to achieve high delivery ratio by selecting the most suitable next hop with new link cost function. Table 1 shows the comparison of the existing protocols and a summary of them. In addition to them, there is still remaining research work to address the other issues such as unbalanced energy consumption.

First, Yousaf et al. proposed Critical data transmission in Emergency with Mobility Support in WBANs (CEMob) [28] to prevent duplicated data transmission by taking priority of packets. In CEMob, two types of communications, single-hop and multi-hop communications, are adaptively selected according to a person’s movement. To be detailed, sensed value at each node is divided into either critical or normal data. In the case of critical data, all nodes send data packets to the sink in single-hop communications to reduce delay at the intermediate node. On the other hand, current value is compared with the previous value prior to transmission in normal data. If two values are identical, they are discarded to prevent resource consumption by duplicated transmission. Otherwise, a data packet is delivered to an advanced node that is defined to have the largest remaining battery. Upon receiving, this advanced node relays the data packet to the sink node. In this work, the advanced node is the normal node but has a larger amount of battery than others. This surplus energy is used to reduce power and resource consumption with the help of data aggregation.

Another consideration for WBAN is to optimize the data rate to reduce resource consumption. This problem was addressed by polling based MAC protocol in [29]. Kin et al. pointed out that temporal disconnection caused by mobility makes link quality worse and leads to changing optimal bit rate frequently. To implement this protocol, seven types of messages and special nodes called hubs need to be defined. Polling is determined by both transmission duration and optimal bit rate after measuring the channel quality.

Finally, unbalanced energy consumption issues caused by mobility were addressed in a way of enhancing the On Increasing Network Lifetime (OINL) routing algorithm. Since original OINL tends to include a large number of intermediate nodes on the path, it suffers from huge energy consumption for data reception as well as aggregation. Thus, M. Sahndhu et al. proposed Balanced Energy Consumption (BEC) [30] to select proper relay nodes with remaining energy. To increase a network lifetime, reactive routing becomes active to transmit critical data when a node’s energy level is below the threshold. In addition, O. Smail et al. addressed the same issue and presented a new routing protocol in [31]. In the proposed scheme, a sink node selects a new forwarder with remaining energy in every round. After a sink node designates forwarder nodes, all sensor nodes transmit data packets to these forwarders, which perform aggregation. Aggregated data is transmitted to the sink node or the next forwarder node. Since a forwarder node is selected in every round according to current energy level, balanced energy consumption is naturally achieved. The proposed scheme operates in a similar way as SIMPLE protocol. Other energy aware protocols, Link-Aware and Energy Efficient protocol for wireless Body Area networks (LAEEBA) and COoperative (CO)-LAEEBA, were introduced in [32]. In LAEEBA, a sensor node broadcasts the packet containing locations of sink nodes, all paths to sink node, own ID, own location, and remaining energy. Upon receiving this packet, a neighboring node updates its routing table accordingly. Like SIMPLE, energy consumption is balanced by rotating forwarder nodes in specific time intervals. The selection of forwarder node is dependent on Cost Function (CF) that is computed by node’s ID, distance, and remaining energy. Based on distributed CF value, each sensor node designates its forwarder as one having the minimum CF value. Fundamentally, CO-LAEEBA operates in the same way as LAEEBA, but a new cooperative node was newly introduced. CO-LAEEBA assumes heterogeneous architecture; therefore, cooperative and sink nodes have higher capacity of data transmission and computing power than normal nodes. While a forwarder is iteratively selected in predetermined intervals for balanced energy consumption in LAEEBA, a cooperative node is continuously updated in CO-LAEEBA. As a person moves, each sensor node on the body has a tendency to have multiple paths to sink nodes as compared to single paths in static situations. In order to utilize multiple paths in an efficient way, normal data is forwarded to a cooperative node, but emergent data is directly passed to the destination node. However, if the energy level of a cooperative node is lower than the threshold, normal data is transmitted to the destination node directly without relaying. The last approach to reducing energy consumption through the relay node was presented in [33]. In this study, direct communication was performed whenever the coordinator moved to the coverage area of the sensor or relay node due. Otherwise, a sensor node needs the help of sensor nodes or relay nodes to transmit data packets to the coordinator. Under this basic principle, there are three cases for the next hop selection. At first, the neighboring node having the shortest distance to the coordinate node is chosen primarily. The next considerable factor is relative velocity. That is, a node having the similar moving velocity to the coordinator is the next candidate. At last, a node with higher residual energy level than the threshold is selected as the next hop.

#### 3.1.3. Protocols for Specific Mobility

The existing specific individual mobility models for WBANs are largely dependent on one application, patient monitoring, which is based on the patient mobility model [34] to generate the movement trace of patient in the hospital. In addition, the few protocols proposed to maintain network topology are as follows.

**Figure 5:**
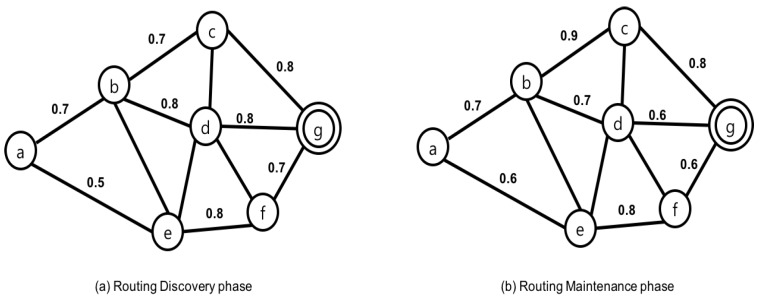
Operations in Reliable Multi-Path Routing.

First, Reliable Multi-Path Routing (RMPR) [35] belongs to reactive protocols. Its operation consists of three major phases: route discovery, route maintenance and data delivery. At the route discovery phase, a sensor node floods the Route Request (RREQ) packet to all neighbors. Intermediate node information and a new metric, String Factor (SF), are added to the packet accordingly. After receiving this RREQ message, a sink node replies with a Route Reply (RREP) packet to establish multiple paths. A source node decides the routing path with the highest SF value for the path. Once the path is established, if link quality on the routing path becomes worse, a path is replaced by one with the highest sum of SF in the route maintenance phase. In this case, multiple paths are used to transmit reply messages. Figure 5 shows the operations of RMPR. In this example, SF is set to a ratio of the received power level between two nodes where node *a* is source and *g* is sink. After RREQ and RREP packets are exchanged, three separate paths are distinguished according to SF value: (i) a − b − c − g = 0.39; (ii) a − b − d − g = 0.44; and (iii) a − e − f − g = 0.28. These values are computed by multiplying SF values of all intermediate nodes along the path. Among them, path (ii) is selected because it has the highest SF value. When SF value varies due to node movement as shown in Figure 5b. SF value on each path is updated as (i) a − b − c − g = 0.5, (ii) a − b − d − g = 0.29, (iii) a − e − f − g = 0.28, respectively. According to the path selection rule, path (i) is selected for packet delivery.

Second, Loose association Implicit reservation Protocol for Mobile WBANs (LIMB) [36] was proposed by assuming that the longer the human stays in the same position, the larger the probability of movement. To implement LIMB MAC protocol, there are two types of nodes: mobile and static node. Braem et al. explained the superframe structure with three phases and association mechanism. In addition, the backbone protocol handles duplicated packets and acknowledgement processing so as not to reach maximum delay. The backbone network operates in one hop communication.

### 3.2. Group Mobility

The group mobility model represents nodes that are moving around the logical center with weak or strong relationships between them. There are some mobility models and communication protocols for group mobility in WBAN. In addition, the group mobility model is largely dependent on the collaboration application; therefore, network architecture such as C-SPINE [5] needs to cooperate with the group mobility model.

#### 3.2.1. Mobility Model

The well known group mobility model is Reference Point Group Mobility (RPGM) [37] that defines the correlation between nodes in the group. In addition, column mobility, pursue and nomadic community model [38] were proposed to model a person’s movement patterns in specific scenarios. In addition, Small World In Motion (SWIM) makes use of social properties to decide the next moving places [39]. N-Body model [40] is another model to extract the social information from real human movement traces by defining friendship and steadiness matrices. Nabi et al. [41] extended the RPGM model by introducing new parameters related to posture with posture selector and global movement models.

Furthermore, as a special case, Random Room Mobility (RRM) [42] was proposed to cover Inter-WBAN and Inter-WBAN mobility models that describe movement of patients in the hospital. In this model, each patient moves to one room according to probability mass function. If a man enters that room, the location of Destination Point (DP) is computed by length, breadth, and height of the room. Upon designating DP, a patient’s speed, pause time and velocity is set. The position information is continually generated until current position reaches the DP where Center Point (CP) is updated according to moving time and velocity of nodes on a patient’s body. In particular, the group mobility model for soccer players was presented in [43]. A new DynaMo model solves the problem of the previous work which is collaborated with image processing and tracking techniques during a match. Finally, the impact of the mobility model is analyzed and compared with the original RPGM from the aspects of throughput and delay.

#### 3.2.2. Protocols for General Mobility

**Figure 6:**
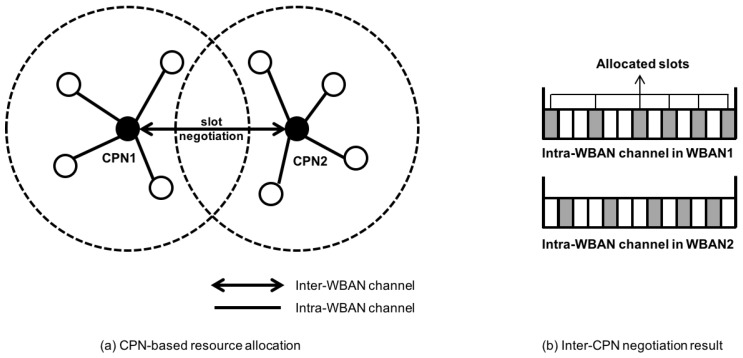
Example of channel allocation in Random Contention-based Resource Allocation.

The Random Contention-based Resource Allocation (RACOON) [44] is a bandwidth control system to support multi Quality of Service (QoS) in MAC layers. It has two important components: Central Processing Node (CPN)-based resource allocation and random contention-based inter-CPN negotiation. Unlike typical WSN, CPN-based protocol has two separate channels. Inter-WBAN channel is used to exchange resource negotiation message while Intra-WBAN channel is for polling and data packets. CPN sends a polling message to each sensor node according to the polling slot. When the coverage of two nodes is duplicated, CPN resource negotiation is performed in the Inter-WBAN channel. In this protocol, different priority and bandwidth are given to each WBAN. Figure 6 illustrates an example of resource allocation in RACOON. In Figure 6a, if two WBANs have duplicate coverage areas, inter-WBAN channel is used to perform CPN resource negotiation. After negotiation, a superframe slot for the Intra-WBAN channel is used for data communications as shown in Figure 6b. As an approach in the MAC layer, Time-Synchronized Channel Hopping (TSCH) can be applied in WBAN to provide collision-free and interference-avoiding communications. In order to reach a steady state in early time without mobility, decentralized time- synchronized channel swapping (DT-SCS) was proposed in [45] to achieve high bandwidth utilization, and connectivity was also achieved.

The other protocol to use group mobility to extend WBAN network lifetime was introduced in [46]. When the coordinate node on the body is intended to deliver packets to access points, intermediate coordinate nodes operate as relay nodes if connection is not available due to a person’s movement. Another disconnection occurs when the energy level of the coordinate node becomes lower than the threshold. In this case, the coordinate node notifies sensor nodes to change the coordinate node. After identifying the coordinate node’s status, a sensor node searches for available coordinate nodes around it. In order to handle mobility in time-efficient ways, the interval of HELLO messages is proportional to mobility speed. This implies that more HELLO messages are generated to update topology information in highly dynamic networks. The factor, HelloP, is computed by the two following Equations (1) and (2), where highmobSpeed represents the highest mobility speed and lowmobSpeed the lowest mobility speed, respectively.

(1) $$HelloP=\left.coverage/mobSpeed,\right.$$

(2) $$mobSpeed=\left.\left(highmobSpeed+lowmobSpeed\right)/2.\right.$$

**Figure 7:**
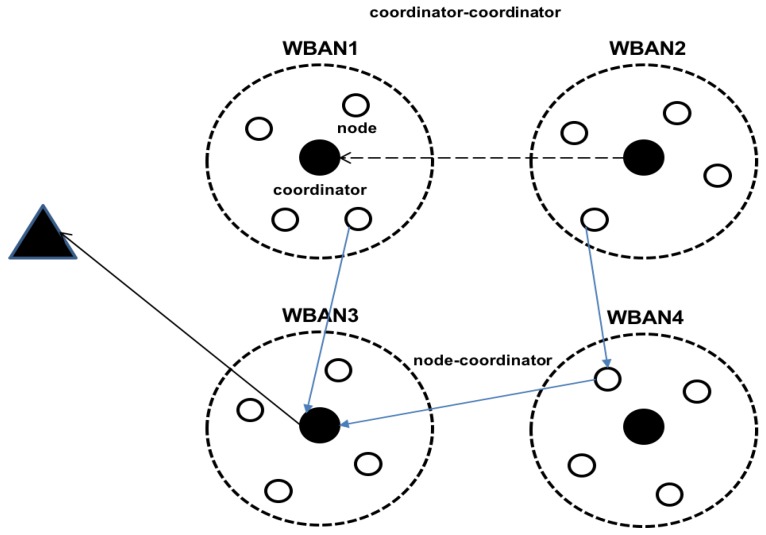
Inter-WBAN communication.

Figure 7 illustrates the scenario for cooperation among four WBANs. When the battery of coordinator nodes in both WBAN1 and WBAN4 is almost drained out, a sensor node in WBAN1 transmits packets to new coordinators in WBAN3 while a node in WBAN2 sends packets to the nearest node in WBAN4 through node–node communications. Consequently, a receiving sensor node in WBAN4 sends packets to coordinators in WBAN3 instead of one in WBAN4, which suffers from low battery power.

For the handover issue, there is a good technical paper [47] to maintain the accessibility of sensor nodes in healthcare wireless sensor networks (HWSN). Caldeira et al. presented main principles and intra-mobility schemes in HWSN. In addition to approaches in WSNs, a fuzzy based handoff mechanism [48] was proposed to switch links between APs for WBANs. In this model, there are two users: Primary Users (PUs) and Secondary Users (SUs). Specific QoS threshold should be guaranteed for PUs. To ensure QoS, fuzzy logic determines whether handoff is performed. This decision is initiated by the level of signal strength. The MAC layer approach without backup server for WBAN was proposed in [49]. As for the handover issue, dynamic channel allocation problem is essential for mission-critical applications; therefore, interference mitigation is important to minimize power consumption and increase the reliability of the system. Thus, Movassgaghi et al. [50] proposed the prediction based algorithm through Smart Channel Assignment (SCA) techniques for unknown dynamics. To accommodate variations in link quality and network topology caused by handover, various mobility scenarios amongst coexisting WBAN were analyzed.

#### 3.2.3. Protocols for Specific Mobility

Until now, protocols for specific mobility focus on soccer players, patients in hospitals, and others. First, a new approach FAtigue MEasurement (FAME) [51] was presented to report the fatigue value for soccer players and soldiers. The protocol assumed RPGM and measure fatigue through vivo sensors on the leg. When the player’s fatigue value exceeds the threshold, its value is transmitted near the base station in direct communications. This protocol can be used to include three different exercising modes for soliders: Soldier Walking Model (SWM), Soldier Slow Running Model (SSRM), and Soldier Fast Running Model (SFRM). Their velocity is set to around 5 km/h, 15 km/h and 24 km/h, respectively. In addition, there are few special vibrational pads attached on the leg to reduce the fatigue.

**Figure 8:**
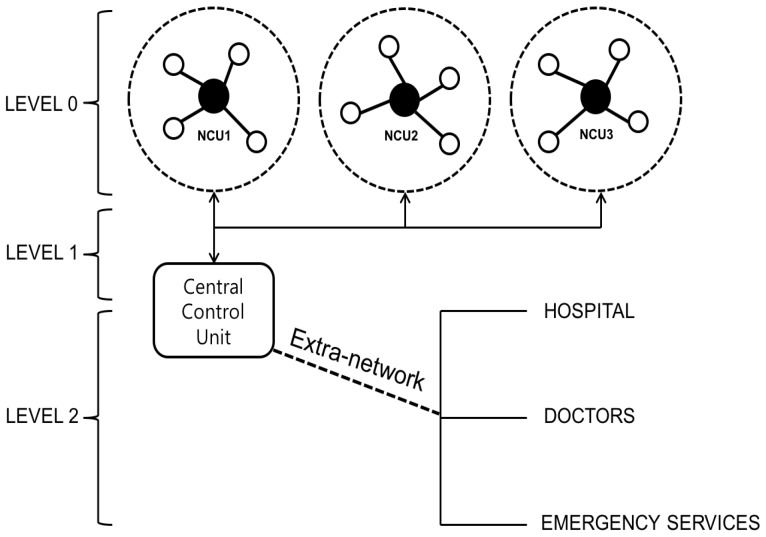
Architecture of Cognitive Radio enabled Wireless Body Area Networks.

Second, two outstanding protocols were presented: Distance-Aware Relaying Energy-efficient (DARE) and Mutual Information based (MI-DARE) [52] for eight patients in the hospital. In DARE, each patient possesses multiple data monitoring sensors, event-driven data monitoring sensors and one Body Relay node (BR), whereas one sink node locates at the center in a hospital room. Both sensor and BR check their alive status. If the measured value is lower than threshold, a sensor node measures the distance between them. Based on this distance, it computes energy consumption cost and transmission delay. Upon completing computations on all sensor nodes, BR aggregates all information and then sends it to the sink. An extended MI-DARE focuses on continuous data monitoring sensor. Especially, in order to prevent multiple transmissions, the value is transmitted only if the difference between two consecutive values are beyond the threshold. In addition to two papers above, Rathee et al. [53] introduced an architecture to combine WBAN MAC protocol and Cognitive Radio Technology without aiming at any specific environments. The proposed architecture consists of three levels. At Level 0, sensor nodes and actuators are embedded into patients. Each sensor node and actuator has different designs in cognitive approach. In addition, Cognitive Radio Technology (CRT) based Network Coordination Units (NCUs) play a role in sink nodes on the body. The functions include data fusion, spectrum sensing, configurable networking, energy efficiency and reliable data routing, in addition to wireless cognitive connectivity with Central Controlling Units (CCUs). Next, Inter-WBAN communications at Level 1 are accomplished by more than two NCUs thats are connected to CCUs. The CCUs are responsible for spectrum sensing, energy efficiency and highly reliable routing and QoS awareness. Finally, CCUs make wireless connections to external hospitals and doctors in Level 2. The architecture is illustrated in Figure 8, where NCUs and CCUs are additionally embedded into existing modules to support CRT.

## 4. Open Issues

Even though lots of researchers addressed the importance of mobility support and proposed new networking technology for WBAN, it is still worthwhile to mention points for further study.

1. Mobility Model for Network Simulator: most of the research works for WBAN include performance evaluation results to prove their suitability through simulation. The mobility model is very important and plays a significant role in evaluating the performance. This indicates that the mobility model should be operated with a network simulator in a cooperative way. Currently, OMNeT++ and NS-2/3 are general frameworks to conduct simulation for WBAN. Therefore, it is demanded to get a free, available WBAN mobility model as an add-on for these frameworks. To meet this requirement, mobility generating tools and analysis tools such as BonnMotion (University of Osnabrück, Osnabrück, Germany) [54] are essential for the mobility model. Furthermore, its analysis should be done to prove the accuracy of the mobility model.
2. Various Mobility Models: Generally, the mobility model is designed to explain the movement pattern of mobile nodes and how their locations change over time according to velocity and direction. Thus, it is desirable for mobility models to emulate the movement pattern of targeted real-life applications in a feasible way. Even though a few applications are now available and deployable nowadays, current mobility models are not enough to describe movement of nodes accurately. Therefore, further research for human behavior is required to enhance mobility models.
  In addition, current mobility models do not take external environments, such as roads in the city for walking and running postures into account; therefore, a combination of several mobility models that describe the moving boundary contributes to improvement of accuracy. One of the candidates is the Manhattan model. In addition, since human behavior is affected by the different situations such as age and living place, they should be taken into account for mobility design.
3. Integration with External System: one of the best sources to develop mobility patterns is to collect traces. If we have any device to track all movements in an efficient way, this will be the best option to get traces. Recently, smartphones have become pervasive so research work for smartphone-based human mobility prediction has been initiated. As Do et al. [55] proposed, it will be a good way to make use of smartphones to predict the movement and develop the mobility models. This is a reason to integrate mobility models with external systems. In addition, WSN, infrastructure for vehicular ad hoc networks and mobile networks can be good sources to collect human mobility, especially for group mobility. Therefore, interfaces to get the demanded information with external systems can accelerate the mobility model research.
4. Network Architecture: Current research work is assumed under homogenous as well as flat network architecture. However, recent routing protocol gradually takes clustering or hierarchical architecture into account as observed in [56]. To catch up with this trend, mobility support should be designed to operate under heterogeneous networks architecture. In this architecture, different mobility patterns need to be applied according to types of nodes. In addition, mobility relationships between two types of nodes is required to integrate them into a system.
5. Security: Contrary to benefitting from external systems, security is one of the important research challenges in WBAN. Furthermore, mobility results in more critical tasks for security because new authentication may be required to new topology, especially in the case of handovers. More seriously, when it comes to additional overhead to handle mobility, it leads to uncertainty of security support on a resource constrained node.

## 5. Conclusions

In this paper, we presented the importance of mobility support in WBAN. As new applications have emerged, the mobility model plays a significant role in protocol design and performance evaluation. We categorized the current research works according to group members as well as specific requirements. Furthermore, we briefly described the main operational procedures of them and compared them to each other. Finally, further research challenges and issues were explained to give guidelines for research trends.

## Figures and Tables

**Table 1:** Comparison of routing protocols.

Protocol	Basic Principle	New Metric or Architecture	Underlying Technology	Target Performance Parameter
OBSFR	Store and forward	Distance to the sink	Flooding	Delay
PRPLC	Store and forward	Link Likelyhood Factor		Delay
DVRPLC	Store and forward	Link Cost Factor		Delay
ETPA	Store and forward	Cost of transmission to neighbor		Energy and temperature
Opportunity Routing	Store and forward	Relay node	RTS/ACK	Energy
RTM-RP	Store and forward	Relay node	CSMA, TDMA	Energy and temperature
M-ATTEMPT	Store and forward	Heterogeneous	TDMA	Energy and delivery ratio
MTR	Store and carry	Meeting probability with sink node		Temperature
